# Paulownin elicits anti-tumor effects by enhancing NK cell cytotoxicity through JNK pathway activation

**DOI:** 10.3389/fphar.2024.1439079

**Published:** 2024-09-04

**Authors:** Eun Sun Park, Yo Sep Hwang, Hyung Won Ryu, Hyang Ran Yoon, Jong-Tae Kim, Jong-Seok Lim, Hee Jun Cho, Hee Gu Lee

**Affiliations:** ^1^ Immunotherapy Research Center, Korea Research Institute of Bioscience and Biotechnology, Daejeon, Republic of Korea; ^2^ Department of Biomolecular Science, KRIBB School of Bioscience, Korea University of Science and Technology (UST), Daejeon, Republic of Korea; ^3^ Natural Research Center, Korea Research Institute of Bioscience and Biotechnology, Cheong-ju, Republic of Korea; ^4^ Department of Biological Science and the Cellular Heterogeneity Research Center, Research Institute of Women’s Health, Sookmyung Women’s University, Seoul, Republic of Korea

**Keywords:** paulownin, NK cells, innate immunity, immunotherapy, JNK

## Abstract

Paulownin, a natural compound derived from *Paulownia tomentosa* wood, exhibits various physiological functions, including anti-bacterial and anti-fungal effects. However, the impact of paulownin on natural killer (NK) cell immune activity remains largely unknown. In this study, we investigated the effect of paulownin on NK cell activity both *in vitro* and *in vivo*, and explored its potential mechanisms. NK-92 cells were used for *in vitro* experiments and a BALB/c mouse model with B16F10 cells injected subcutaneously were used for *in vivo* anti-tumor analysis. We found that paulownin enhanced the cytolytic activity of NK-92 cells against leukemia, human colon, and human lung cancer cell lines. Paulownin treatment increased the expression of the degranulation marker protein CD107a and cytolytic granules, including granzyme B and perforin in NK-92 cells. Moreover, these enhancements of cytotoxicity and the expression of cytolytic granules induced by paulownin were also observed in human primary NK cells. Signaling studies showed that paulownin promoted the phosphorylation of JNK. The increased perforin expression and elevated cytotoxic activity induced by paulownin were effectively inhibited by pre-treatment with a JNK inhibitor. *In vivo* studies demonstrated that the administration of paulownin suppressed the growth of B16F10 melanoma cells allografted into mice. Paulownin administration promoted the activation of NK cells in the spleen of mice, resulting in enhanced cytotoxicity against YAC-1 cells. Moreover, the anti-tumor effects of paulownin were reduced upon the depletion of NK cells. Therefore, these results suggest that paulownin enhances NK cell cytotoxicity by activating the JNK signaling pathway and provide significant implications for developing new strategies for cancer immunotherapy.

## 1 Introduction

Conventionally, tumor treatment involves surgery, chemotherapy, radiotherapy, immunotherapy, and a combination of these treatments (Debela et al., 2021). Cancer immunotherapy activates the individual’s natural immune system to fight cancer. Different types of immune cells, including natural killer (NK) cells, T lymphocytes, B lymphocytes, and dendritic cells (DCs), have various clinical applications such as immune checkpoint inhibitors, cytokine therapies, engineered T cells, and cancer vaccines (Riley et al., 2019; Zhang and Zhang, 2020). Moreover, immunotherapy has shown great potential for treating multiple types of cancer and has demonstrated satisfactory outcomes in many patients with cancer. Although many immunotherapeutic strategies primarily focus on T cells, NK cells are rapidly becoming an attractive option for therapeutic interventions.

NK cells are cytotoxic immune lymphocytes that are important for early host defense by lysing cancer cells without prior stimulation. NK cells also cooperate with other immune cells in the adaptive immune response in cancer environments (Zhang and Zhang, 2020; Rossi et al., 2022). NK cells can be derived from various sources, including peripheral blood, umbilical cord blood, and induced pluripotent stem cells. They constitute approximately 10% of the peripheral blood components and are found in various organs such as the spleen, lung, and liver (Rossi et al., 2022). Several new principles have been developed to activate NK cell function because of the critical role of NK cells in immune responses. These approaches include adoptive cell transfer, cytokine therapies, monoclonal antibodies targeting activating and inhibitory receptors, allogeneic and autologous NK cell treatments, and gene-edited CAR-NK cell therapy (Shimasaki et al., 2020; Du et al., 2021) Lytic granule exocytosis is one of the key mechanisms underlying NK cell killing strategies. Activated NK cells release perforin, which opens pores on the target surface, allowing granzymes to enter and induce apoptosis in target cells via immunological synapse (Krzewski and Coligan, 2012).

Natural products isolated from plants or animals, which have various biological properties, have become attractive materials for cancer immunotherapy (Dong et al., 2022). These natural products can regulate immune responses while killing tumor cells through several key mechanisms, such as STAT3, Akt, and MAPK signaling pathways (Yu et al., 2016; Han et al., 2019; Huo et al., 2022). Furthermore, some natural products have shown great potential in modulating the function of NK cells (Deng et al., 2020). For example, the combination treatment of asiatic acid and naringenin upregulates natural killer cytotoxicity against cancer in mouse models (Lian et al., 2018). Moreover, artemisinin extracted from the sweet wormwood plant enhanced NK cytotoxicity by stimulating the ERK 1/2 and Vav-1 pathways (Houh et al., 2017). Paulownin is a natural compound found in the wood of *Paulownia tomentosa* Steud (Schneiderova and Smejkal, 2015). Different parts of the paulownia tomestosa Steud had been utilized in remedies for various diseases due to their imunomodulatory properties (Schneiderova and Smejkal, 2015). Specifically, polysaccharides derived from flowers of Paulownia tomentosa are known to enhance immune responses by stimulating innate immunity (Chen et al., 2021). Additionally, β-sitosterol glycoside from Paulownia leaves have been shown to modulate the immune system by promoting the production of Th1-dominant cytokines though the activation of CD4 + T cell (Lee et al., 2007). Paulownin has anti-bacterial activity against *Pseudomonas putida* (Rao et al., 2012) and anti-fungal activity against *T. versicolor* (Kawamura and Ohara, 2005). Although some studies have reported that paulownin exhibits cytotoxic effects against human lung and breast cancer cell lines *in vitro* (Huang et al., 2014; Reis et al., 2022), its role in modulating anti-cancer immune activity, particularly in relation of NK cell cytotoxicity, remains unclear.

In this study, we investigated the anticancer effect of paulownin using a mouse melanoma model with B16F10 cells and explored its potential mechanisms in NK-92 cells. Our findings demonstrate the novel function of paulownin as an immune modulator for anti-tumor therapy.

## 2 Materials and methods

### 2.1 Chemicals and reagents

Paulownin was obtained from ChemFaces (Wuhan, China) and dissolved in dimethyl sulfoxide (DMSO; Sigma-Aldrich, St. Louis, United States). The JNK inhibitors SP600125 purchased from Chemcruz (Dallas, USA).

### 2.2 Cell lines and culture

NK-92 cells were cultured in α-MEM (Welgene, Gyeongsan-si, Republic of Korea) supplemented with 12.5% FBS (Gibco, United States), 12.5% horse serum (Gibco), 0.2 mM inositol (Sigma-Aldrich), 0.02 mM folic acid (Sigma-Aldrich), 0.1 mM 2-mercaptoethanol (Sigma-Aldrich), and 20 ng/mL IL-2 (PeproTech, United States). SW480 and HT-29 cell lines were cultured in DMEM medium (Welgene) supplemented with 10% FBS and K562, A549, PC-9, and YAC-1 cell lines were maintained in RPMI 1640 medium (Welgene) supplemented with 10% FBS and all cell lines were obtained from the American Type Culture Collection (ATCC, Manassas, VA, United States). Human peripheral blood NK cells were purchased from Stemcell Technologies (Vancouver, BC, Canada). Primary NK (pNK) cells were expanded using the ImmunoCult NK cell Expansion kit following the manufacturer’s instructions. pNK cells were maintained in RPMI with 10% FBS adding 40 ng/mL IL-2 and 20 ng/mL IL-15 (PeproTech). The cells were maintained at 37°C under 5% CO2.

### 2.3 Cell cytotoxicity assay

The cytotoxicity of NK cells was loaded with calcein-acetoxymethyl (AM) (Invitrogen, Carlsbad, United States) release assay and CytoTox 96 Non-Radiative cytotoxicity assay. NK cells were treated with paulownin under certain conditions. For calcein-AM assay, target cells were stained with 10 μM calcein-AM in RPMI serum-free media for 1 h and co-incubated with effector cells. After a certain incubation time, 100 μL of the supernatant was harvested into a black plate. The absorbance was measured using a microplate reader at the excitation/emission wavelength of 485/530 nm. The percentage of cell lysis was calculated by the following formula: calcein- AM release = [(experimental release–minimum release)/(maximum release–minimum release)] x 100. For lactate dehydrogenase (LDH) assay, each target cell was co-cultured with effector cells for 24 h. After incubation, 50 μL of supernatant was collected into a new plate, and CytoTox96 reagent (Promega, Madison, WI, United States) was added and reacted for 30 min in the dark. Next, stop solution was added to each well, and the absorbance was measured using a microplate at 490 nm. The percentage of specific lysis was calculated using the following formula: [(experimental – spontaneous release of effector cells – spontaneous release of target cells)/(maximum release- spontaneous release)].

### 2.4 Degranulation assay

NK cells treated with vehicle or paulownin were co-incubated with target cells. FITC-anti-CD107a (Biolegend, San Diego, CA, United States) was added and then incubated for the indicated time at 37°C. Cells were stained with APC anti-CD56 antibodies and CD107a expression on NK cell was measured using a FACSverse (BD Biosciences) and analyzed using Flowjo software (BD Biosciences).

### 2.5 Quantitative RT-PCR (qRT-PCR)

Total RNA was extracted from cells using RNA extraction kit (Biofact, Korea). cDNA was synthesized from total RNA using oligo dT primer and GoScriptTM Reverse Transcription System (Promega, Madison, WI, United States). qRT-PCR was performed using AccuPower 2X Greenstar^TM^ qPCR Master Mix (Bioneer) and StepOnePlus Real-Time PCR (Thermo Fisher Scientific). Gene expression was calculated by 2^−ΔΔCt^ method. All samples were tested in triplication. β-actin was used as endogenous control. The primers sequences are listed as follows: β-actin 5′-CAA​ACA​TGA​TCT​GGG​TCA​TCT​TCT​C-3′ and 5′-GCT​CGT​CGT​TCG​ACA​ACG​GCT-3′; NKp46 5′-AGA​ATC​TCC​TTC​GGA​TGG​GC-3′ and 5′-GGT​CCA​ACA​CAG​AGC​TCA​CG-3′; NKp44 5′-TAC​CCA​AAA​AGC​CAC​CTG​CC-3′ and 5′-GTG​TGT​TCA​TCA​TCA​TCA​TCG​CT-3′; NKp30 5′-TTT​CCT​CCA​TGA​CCA​CCA​GG-3′ and 5′-GGA​CCT​TTC​CAG​GTC​AGA​CAT​T-3′; NKG2D 5′-GTT​ACT​GTG​GCC​CAT​GTC​CT-3′ and 5′-AGA​AGG​CTG​GCA​TTT​TGA​GA-3′; Perforin 5′-ATG​TAA​CCA​GGG​CCA​AAG​TCA-3′ and 5′-GTG​CCG​TAG​TTG​GAG​ATA​AGC-3′; Granzyme B 5′-GCA​GAT​GCA​GAC​TTT​TCC​TTC-3′ and 5′-CAC​AGG​GAT​AAA​CTG​CTG​GGT-3′.

### 2.6 Western blot analysis

The total cell was lysed in radioimmunoprecipitation buffer containing phosphatase inhibitors and protease inhibitors. The protein concentration was quantified by a BCA protein assay kit (Intron Biotechnology, Kyonggi-do, Republic of Korea). The equal amounts of lysates were separated by 10% or 12% SDS-PAGE and transferred to a PVDF (Bio-Rad, Hercules, CA, United States) membrane using a Trans-Blot^®^ Turbo™ Transfer pack (Bio-Rad). The membranes were incubated with 5% skim milk for 2 h, and then, primary antibodies were incubated overnight at 4°C. Next, appropriate HRP-conjugated secondary antibodies were incubated for 40 min at room temperature. The membranes were visualized using a chemiluminescent HRP substrate (Milipore, Billerica, MA, United States).

### 2.7 Flow cytometry analysis

NK cells were blocked with PBS containing 2% BSA for 1 h at 4°C. NK activating receptors were stained with PE-anti-NKp46, PE-anti-NKp44, APC-anti-NKp30, and PE-anti-NKG2D for 1 h at 4°C. For cytolytic granule analysis, NK cells were permeabilized using Fixation/permeabilization buffer (BD Bioscience, Franklin Lakes, United States) for 30 min and PE-anti-perforin and FITC-anti-granzyme B stained for 30 min at 4°C. The samples were examined using FACSverse (BD Biosciences) and analyzed using Flowjo 10 software (BD Biosciences).

### 2.8 Cell Counting Kit (CCK)-8 viability test

The viability of NK cells was assessed using Cell Counting Kit-8 (Dojindo molecular technologies, Kumamoto, Japan) assay. NK cells were plated at 96-well plates (2 × 10^4^ cells/well) and treated with different concentrations of paulownin for 24 h and 48 h. Following incubation, CCK-8 reagent was added to each well for 3 h The absorbance was detected at a wavelength of 450 nm using a microplate reader (Bio Rad).

### 2.9 Animal experiments

Six-week-old Balb/c mice were obtained from DBL (Korea) and housed in a pathogen-free room at a KRIBB animal care facility. All animal studies were performed according to the guidelines approved by the KRIBB Institutional Animal Care and Use Committee (KRIBB-AEC-22056). Mice were housed in temperature- and air-conditioned sterile plastic cages under a 12/12-h light/ dark cycle. For the anti-tumor assay, B16F10 cells were injected subcutaneously (s.c.) into the right flanks of mice. After day 18, the mice were sacrificed, and tumor weight and volume were measured. Tumor volume for each mouse was calculated using the following formula: (major axis) x (minor axis) x height x 0.52. Splenocytes were isolated from the spleen and lysed by RBC lysis buffer (Biolegend). The NK cell population was determined by flow cytometry and cytotoxicity of splenocytes against YAC-1 cells analyzed by the LDH assay.

### 2.10 Statistical analysis

Each experiment was performed at least thrice and analyzed using GraphPad Prism 0.4 (Dotmatics). Data are presented in terms of the mean ± standard deviation and analyzed using a one-way ANOVA or Student’s t-test. P value of <0.05 (*), <0.01 (**), <0.001 (***), or <0.0001 (****) were considered statistically significant.

## 3 Results

### 3.1 Paulownin enhances the cytotoxicity of NK-92 cells against human cancer cells

**FIGURE 1:**
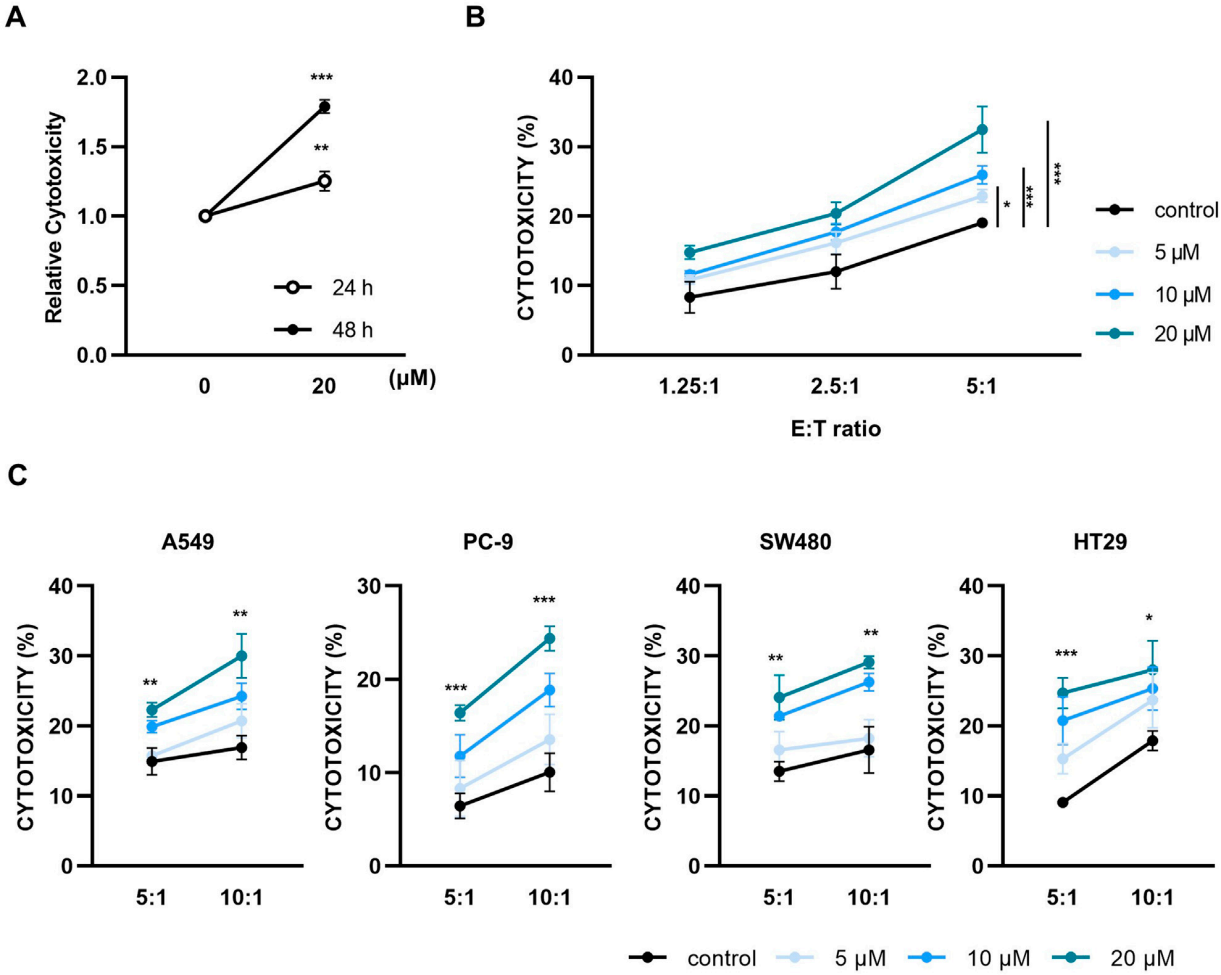
Paulownin enhances the cytotoxicity of NK-92 cells. **(A)** NK-92 cells were treated with 20 μM for 24 h or 48 h and then cytotoxicity assays were performed with K562 cells at a 5:1 ratio using a calcein-AM release assay. **(B)** NK-92 cells were treated with paulownin (0, 5, 10 and 20 μM) for 48 h. The cytotoxicity of NK-92 cells against K562 cells was analyzed by different E: T ratios (1.25:1, 2.5:1, and 5:1) for 2 h **(C)** NK-92 cells were treated with the indicated dose of paulownin and mixed with A549, PC-9, SW480, and HT29 cells. The cytolytic effects of NK-92 cells were evaluated by LDH release assay. The results represent the mean ± SD of three experiments (*p < 0.05, **p < 0.01, ***p < 0.001, ****p < 0.0001 by Student’s t-test, n = 3).

NK-92 cells were treated with various concentrations of paulownin (0, 2.5, 5, 10, 20, 40, 80, and 160 μM) for 24 and 48 h, and were subjected to the CCK-8 assay. Paulownin reduced NK cell viability at concentrations above 80 μM, but had no effect at concentrations below 40 μM (Supplementary Figure S1). Therefore, concentrations below 40 μM were used for subsequent experiments. Next, we evaluated the cytolytic activity of NK-92 cells treated with paulownin against a human lymphoma cell line, K562, using the calcein-AM assay. After 24 or 48 h of treatment with paulownin, NK-92 cells were co-incubated with K562 cells for 2 h at an effector-to-target (E:T) ratio of 5:1. The cytolytic activity of NK cells after 48 h of treatment showed higher cytotoxicity than after 24 h of treatment (Figure 1A). The cytotoxicity of NK-92 cells after 48 h of paulownin treatment was detected using calcein-am assay in dose-dependent at various E:T ratios (Figure 1B). We further investigated whether paulownin enhanced NK cell cytotoxicity against various types of human solid tumor cells, including lung cancer cell lines (A549 and PC-9) and colon cancer cell lines (SW480 and HT-29). NK-92 cells were treated with paulownin (0, 5, 10, 20 μM) and co-incubated with A549, PC-9, SW480, and HT-29 cells for 24 h. NK cytotoxicity was measured using the LDH assay. Pre-treatment with paulownin enhanced the cytolytic activity of NK-92 cells against all tumor cell lines (Figure 1C). Altogether, these findings indicate that paulownin promotes the cytotoxicity of NK cells against various types of cancers.

### 3.2 Paulownin promotes the expression of cytolytic granules in NK-92 cells

**FIGURE 2:**
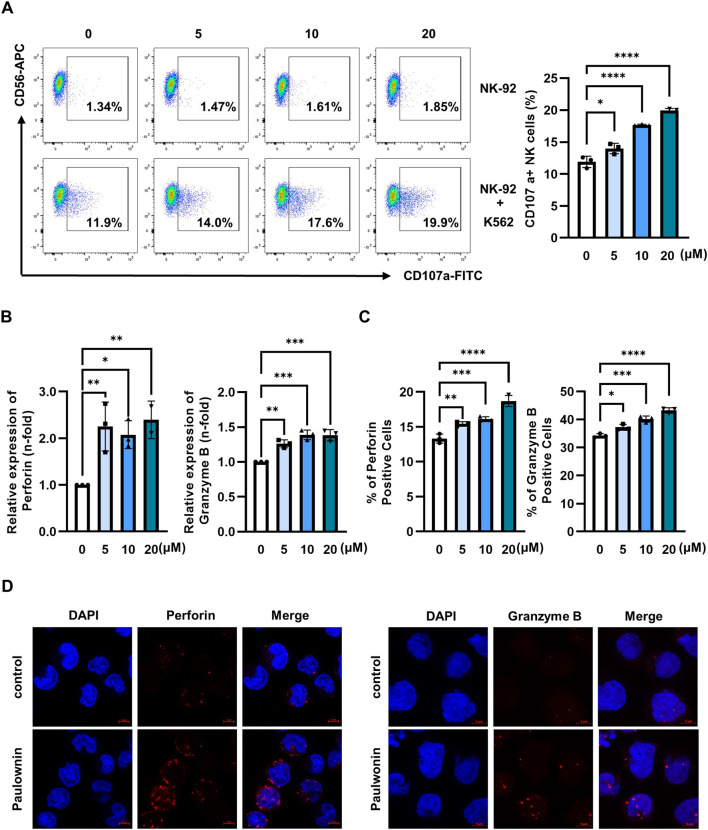
Paulownin stimulates cytolytic granule exocytosis in NK-92 cells. **(A)** NK-92 cells were treated with paulownin (0, 5, 10, and 20 μM) for 48 h. Subsequently, NK-92 cells were co-incubated with K562 cells at a 1:1 ratio for 2 h and stained with fluorescein isothiocyanate (FITC)-conjugated mouse anti-human CD107a antibody. Frequencies of CD56+ CD107a+ expression on NK cells were analyzed using Flow cytometry. Representative results are shown as dot plots and the average percentage of CD56+ CD107a+ expression is presented as statistical bar charts. NK-92 cells were treated with paulownin (0, 5, 10 and 20 μM) for 48 h. **(B)** The expression of mRNA level of perforin and granzyme B of NK-92 cells were evaluated by qPCR **(C)** Protein levels of perforin and granzyme B of NK-92 cells were determined by flow cytometry, with the numbers indicating the percentage of positive cells. **(D)** NK-92 cells were treated with or without 20 μM paulownin for 48 h. The cells were attached to PLL-coated slides. Then, slides were fixed, permeabilized, and stained with Alexa Flour 647-conjugated anti-perforin (Red) or Alexa Flour 647- conjugated anti- granzyme B (Red) along with 4′-6-Diamidino-2-phenylindole (DAPI, blue). Representative images are shown. The results represent the mean ± SD of three experiments (*p < 0.05, **p < 0.01, ***p < 0.001, ****p < 0.0001 by Student’s t-test, n = 3).

The cytotoxicity of NK cells is regulated by multiple mechanisms, such as the balance of inhibitory and activating receptors and cytolytic granules (Park et al., 2022; Chang et al., 2014). Therefore, we first analyzed the expression of major NK activating receptors, including NKp30, NKp44, NKp46, and NKG2D. Paulownin did not affect the mRNA and protein levels of these activating receptors (Supplementary Figure S2). Given the critical role of cytolytic granules in NK activity (Krzewski and Coligan, 2012; Alter et al., 2004), we investigated the effect of paulownin on the surface expression of CD107a in NK cells. NK-92 cells were treated with 5, 10, and 20 μM of paulownin and co-incubated with K562 cells for 2 h. Paulownin treatment enhanced CD107a surface expression in NK cells in a dose-dependent manner (Figure 2A). This enhancement by paulownin was also confirmed when NK-92 cells were co-incubated with A549, PC-9, SW480, and HT-29 cells (Supplementary Figure S3). Next, we determined whether paulownin increased the expression of cytolytic granules, such as perforin and granzyme B. The mRNA and protein levels of perforin and granzyme B were determined by real-time PCR (Figure 2B) and flow cytometry analysis (Figure 2C; Supplementary Figure S4A), respectively. Paulownin treatment sharply increased the expression level of perforin and slightly increased the expression of granzyme B. Furthermore, strong perforin and granzyme B staining was observed in paulownin-treated NK-92 cells compared to untreated cells (Figure 2D). Collectively, these findings suggest that paulownin may enhance the cytotoxicity of NK cells by elevating the expression of cytolytic granules.

### 3.3 Paulownin activates NK cells through the JNK signaling pathway

**FIGURE 3:**
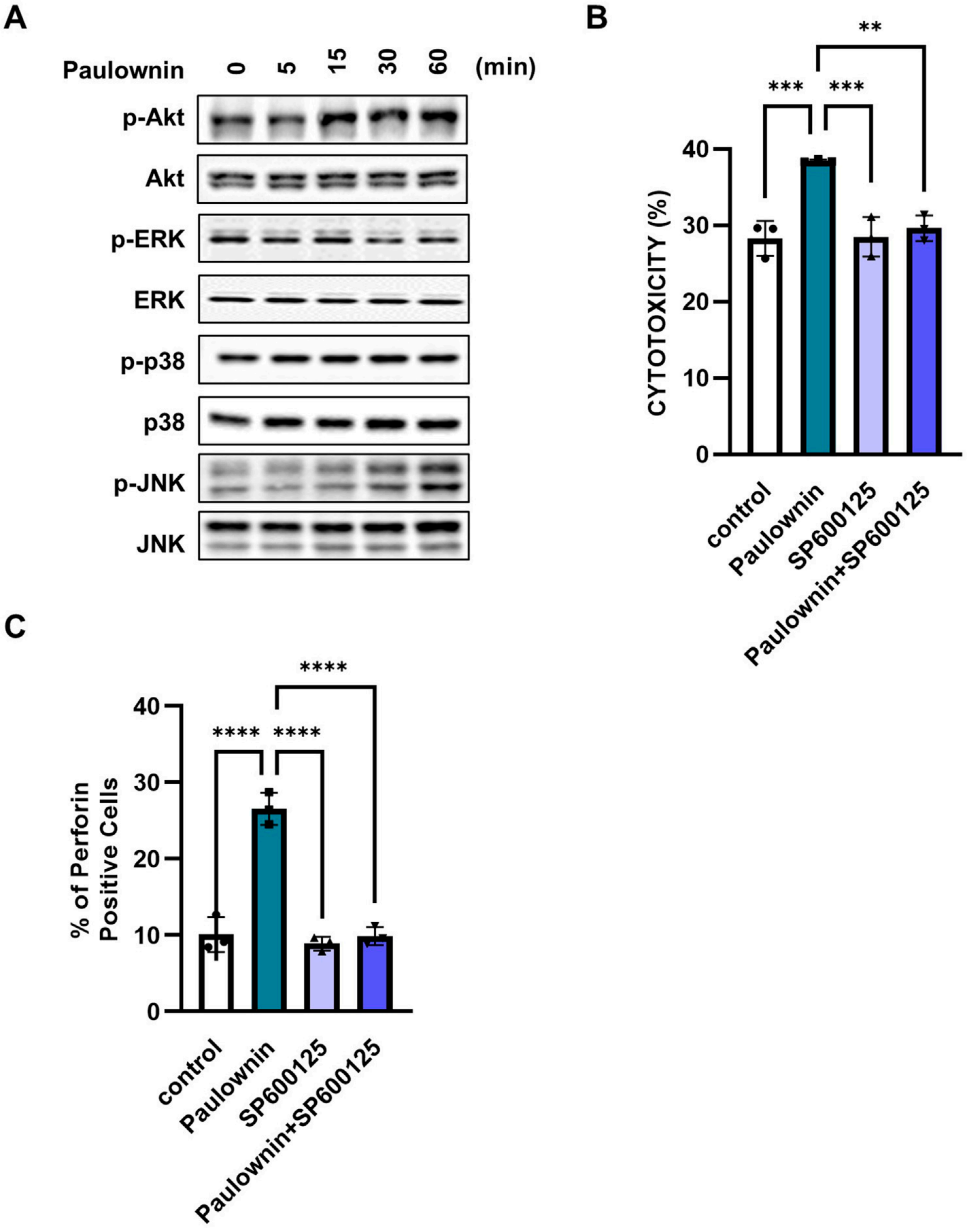
Paulownin activates NK cells through the JNK signaling pathway. NK-92 cells were stimulated with paulownin (20 μM) for 5, 15, 30, or 60 min. **(A)** Expression levels of total and phosphorylated Akt and MAPKs were analyzed by Western blotting. **(B)** NK-92 cells were treated with paulownin only, JNK inhibitor (SP00125) only, or both paulownin and SP00125. The cytotoxicity of NK-92 cells was assessed against K562 cells at a 5:1 ratio. **(C)** The expression of perforin was analyzed using FACS. The results represent the mean ± SD of three experiments (*p < 0.05, **p < 0.01, ***p < 0.001, ****p < 0.0001 by Student’s t-test, n = 3).

Several reports have shown that the cytotoxicity of NK cells is regulated by MAPK and AKT signaling pathways (Yamamoto et al., 2009; Lastwika et al., 2016; Noh et al., 2015). Since paulownin increased NK cell cytotoxicity and the expression of cytolytic granules, we investigated whether paulownin affected the activity of these kinases. NK-92 cells were treated with 20 μM paulownin for indicated times, and Western blot analysis was performed. The results show that paulownin strongly increased the phosphorylation of JNK, but it did not affect the phosphorylation of Akt, ERK, and p38 (Figure 3A). Next, we verified whether paulownin-mediated JNK phosphorylation is responsible for the enhanced cytotoxicity. We treated cells with or without paulownin and with or without the JNK inhibitor. Upregulated cytotoxicity induced by paulownin was reversed by the JNK inhibitor, while the JNK inhibitor alone did not affect the cytolytic activity of NK-92 cells (Figure 3B). Additionally, the JNK inhibitor reduced paulownin-mediated expression of perforin (Figure 3C; Supplementary Figure S4B). These results suggest that paulownin promotes NK cell cytotoxicity by activating the JNK signaling pathway.

### 3.4 Paulownin increases the cytotoxicity of primary human NK cells

**FIGURE 4:**
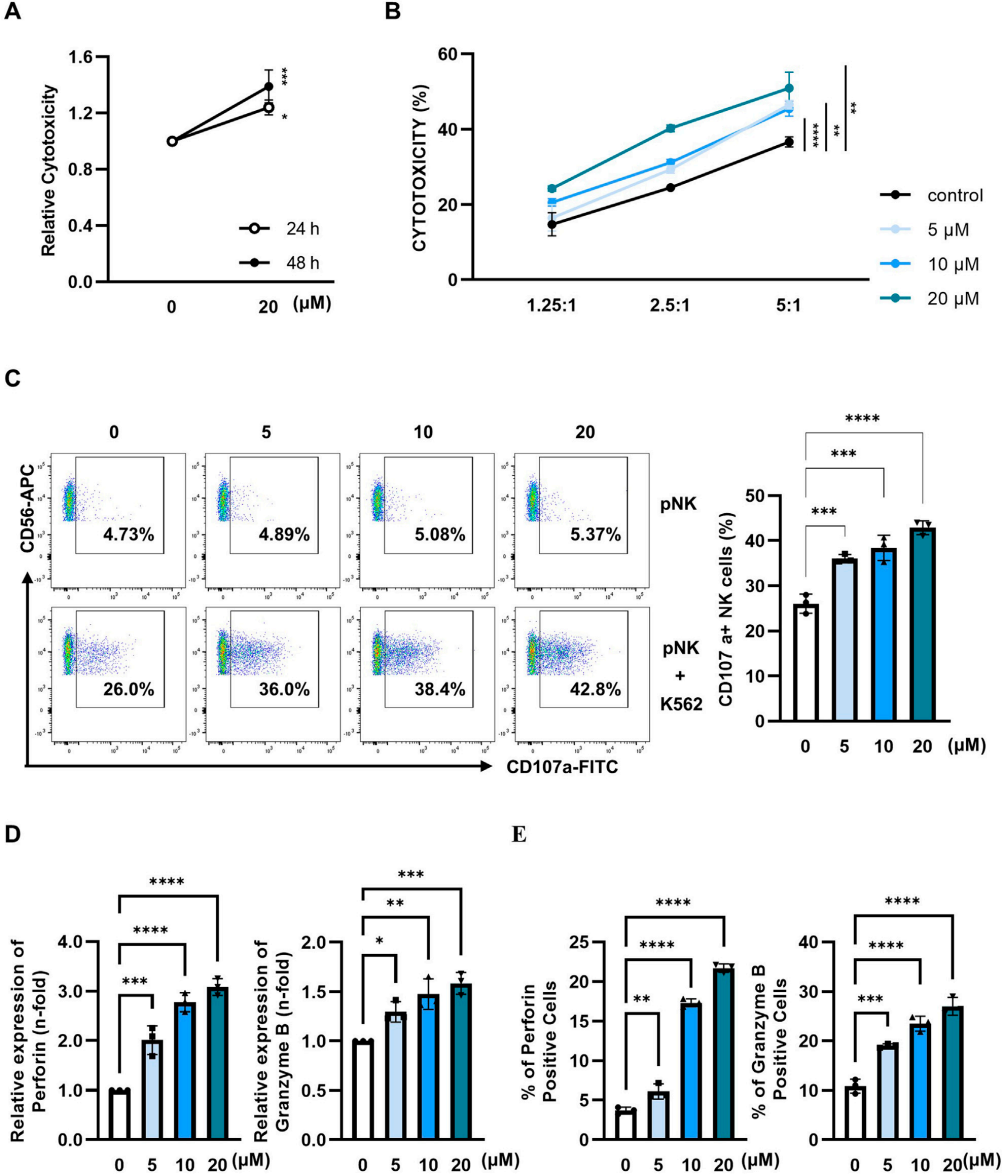
Paulownin enhances the cytotoxicity of primary NK cells. **(A)** pNK cells were treated with 20 μM paulownin for 24 h or 48 h The cytotoxicity of pNK cells toward K562 cells was evaluated using a calcein-AM release assay. K562 cells were used as target cells at an E: T ratio of 5:1 for 1 h **(B)** pNK cells were treated with paulownin for 48 h. The cytotoxicity of pNK cells against K562 cells was analyzed by different E: T (1.25:1, 2.5:1, and 5:1) ratios for 1 h. **(C)** Paulownin pretreated pNK cells were co-incubated with K562 cells at a 1:1 ratio for 1 h and stained with fluorescein isothiocyanate (FITC)-conjugated mouse anti-human CD107a antibody. Frequencies of CD107a expression on NK cells was analyzed using flow cytometry. Representative result shown as dot plot and average percentage of CD107a+ expression shown as statistical bar charts. pNK cells were treated with paulownin (0, 5, 10, and 20 μM). **(D)** The mRNA level of perforin and granzyme B of pNK cells were evaluated by qRT-PCR and **(E)** protein levels of perforin and granzyme B of pNK cells were determined by flow cytometry. Numbers indicate the percentage of positive target cells. The results represent the mean ± SD of three experiments (*p < 0.05, **p < 0.01, ***p < 0.001, ****p < 0.0001 by Student’s t-test, n = 3).

Next, we evaluated whether paulownin also affected the cytolytic activity of primary human NK (pNK) cells. pNK cells were treated with various concentrations of paulownin for 24 or 48 h. Cell viability decreased after treatment with 40 μM paulownin for 48 h (Supplementary Figure S5). pNK cells were treated with paulownin for 24 or 48 h and co-incubated with K562 cells at a 5:1 ratio. The results showed that cytotoxicity increased in pNK cells treated with paulownin for 48 h (Figure 4A). The enhancement of pNK cell cytolytic activity by paulownin was confirmed at various E:T ratios (Figure 4B). We also investigated whether paulownin affected the expression of cytolytic granule markers, CD107a, perforin, and granzyme B, in pNK cells. Paulownin upregulated the number of CD107a-positive pNK cells when co-cultured with K562 cells (Figure 4C). Furthermore, mRNA and protein levels of perforin and granzyme B increased in paulownin-treated pNK cells compared with the untreated group (Figures 4D, E; Supplementary Figure S4C). These results indicate that paulownin may promote the expression of cytolytic granule marker proteins and enhance the cytolytic activity of pNK cells.

### 3.5 Paulownin inhibits tumor growth by increasing NK cytotoxicity in mice

**FIGURE 5:**
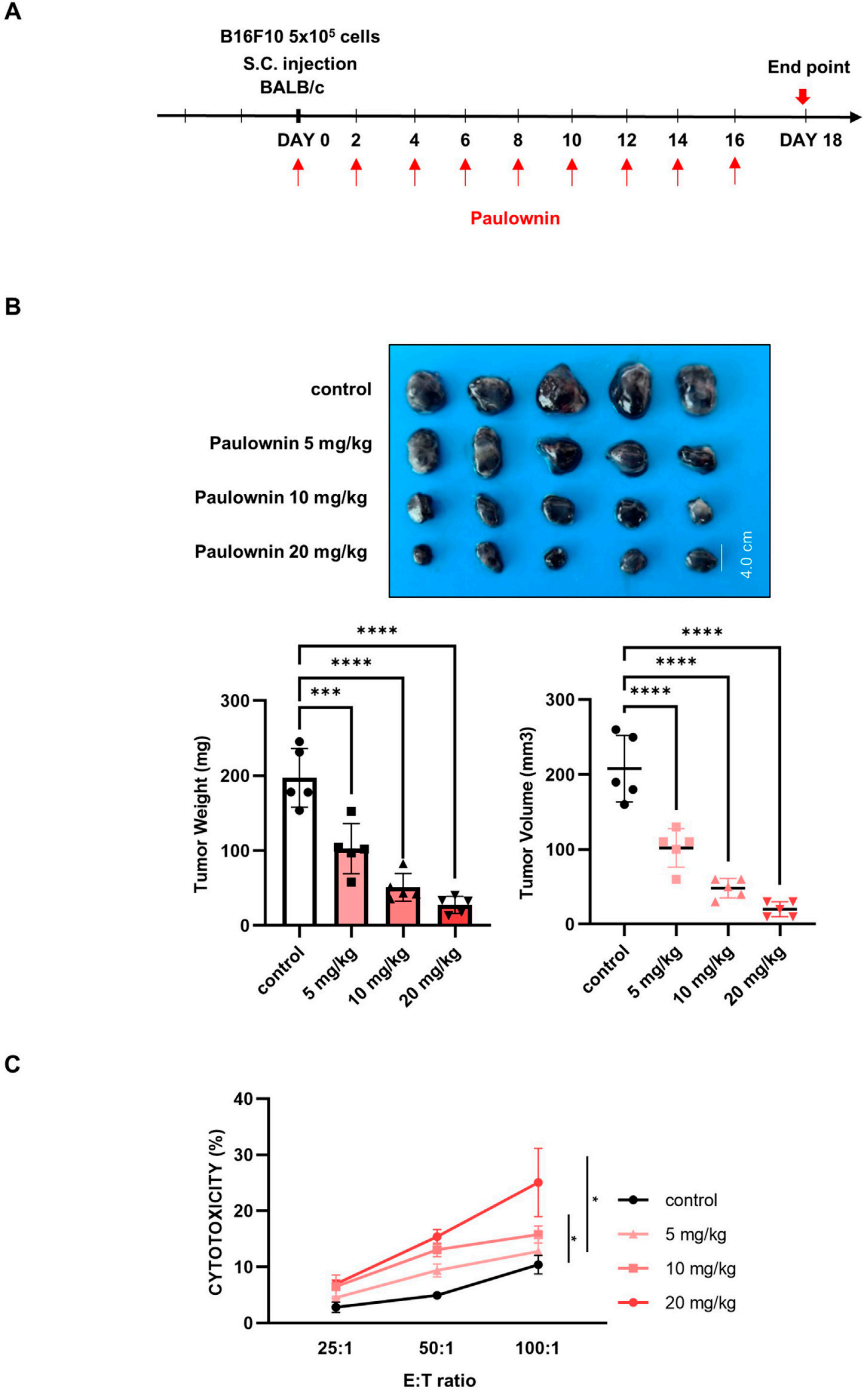
Paulownin inhibited the growth of B16F10 melanoma cancer allograft in Balb/c mice. **(A)** The experimental scheme to detect proper paulownin concentrations. B16F10 cells (5 × 10^5^) were subcutaneously injected into Balb/c mice. Paulownin was injected intraperitoneally every 2 or 3 days. The tumor volume and weight were measured after dissection on day 18 after injection of melanoma cells. **(B)** The tumor image and a summary graph of statistical bar showing each group of tumor volume and weight. **(C)** Splenocytes isolated form balb/c mice were co-cultured with YAC-1 target cell at 25:1, 50:1, or 100:1 for 24 h. The results represent the mean ± SD of three experiments (*p < 0.05, **p < 0.01, ***p < 0.001, ****p < 0.0001 by Student’s t-test, n = 5).

We examined whether paulownin affects NK cell-mediated anti-tumor activity *in vivo*. Mice were subcutaneously implanted with B16F10 melanoma cells and injected with 0, 5, 10, and 20 mg/kg of paulownin (Figure 5A). Tumor volume and weight in mice treated with 5, 10, and 20 mg/kg were significantly lower than those in the control group (Figure 5B), suggesting that paulownin inhibited tumor growth *in vivo*. To verify the cytotoxicity of NK cells in these mice, spleenocytes were isolated from the spleen of each group and co-cultured with YAC-1 target cells for 24 h. Paulownin significantly enhanced the cytolytic activity of splenic NK cells in a dose-dependent manner compared with the control group (Figure 5C).

**FIGURE 6:**
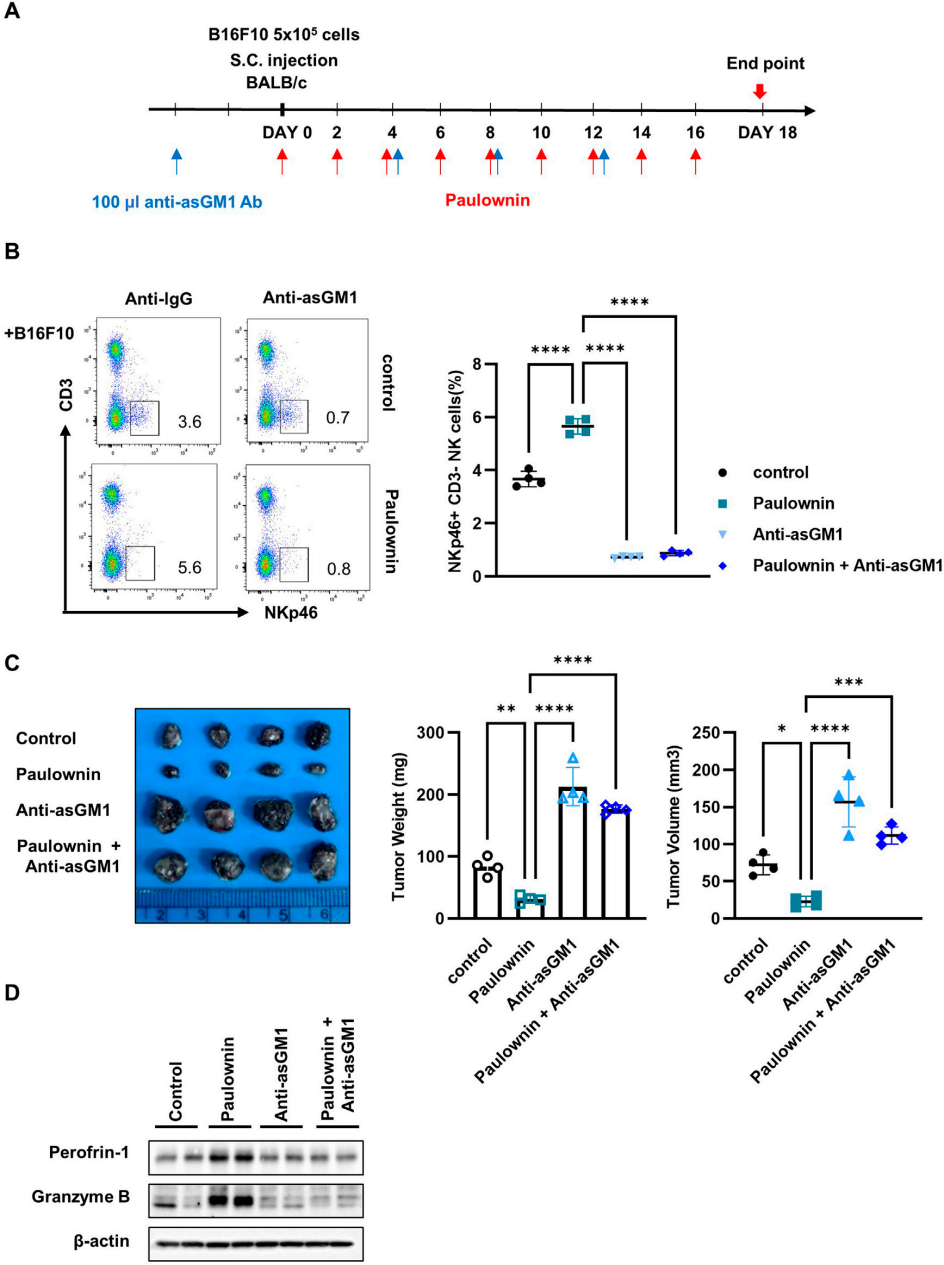
NK depletion abolished anticancer effect of paulownin in mice. **(A)** Overall scheme to investigate the NK activity-inducing effect of paulownin. Balb/c mice were subcutaneously injected with 5 × 10^5^ B16F10 cells and then received either paulownin or vehicle for 2 or 3 days with or without anti-asialoGM1 (100 μL) injection. **(B)** CD3- NKp46 + cells in the spleen of each group were measured with flow cytometry. The left panel shows as dot plot and the right panel shows as graph. **(C)** The tumor volume and weight were measured after dissection on day 18 after the injection of B16F10 cells. **(D)** The expression of Perforin and Granzyme B of spleenocytes was analyzed using Western blot. The results represent the mean ± SD of three experiments (*p < 0.05, **p < 0.01, ***p < 0.001, ****p < 0.0001 by Student’s t-test, n = 4).

To confirm that the anti-tumor effect of paulownin was mediated by NK cells, we divided mice into four groups (n = 4): vehicle, 20 mg/kg of paulownin, 100 µL of Anti-asialo GM1 (asGM1), and 20 mg/kg of paulownin plus 100 µL of anti-asGM1. The experimental schedule is shown in Figure 6A. Mice were sacrificed after 18 days, and the frequency of CD3-NKp46+ cells was detected by flow cytometry. Compared to the control group, the CD3-NKp46+ cell population was enhanced in paulownin-administered mice, which was abolished by anti-asGM1 injection (Figure 6B). Tumor volume and weight were reduced in paulownin-treated mice compared to the vehicle control. The anti-tumor effect of paulownin was abolished by anti-asGM1-mediated depletion of NK cells (Figure 6C). The protein levels of perforin and granzyme B in spleenocytes were analyzed by Western blotting. Both perforin and granzyme B levels increased in the paulownin-injected group compared to the control group. Moreover, their levels in the NK depletion group and the combination of paulownin and NK depletion group were reduced compared to the paulownin-only group (Figure 6D). Altogether, these results indicate that paulownin enhances the cytotoxic potential of activated NK cells against B16F10 cells *in vivo*.

## 4 Discussion

Paulownin is a natural compound found in the wood of *Paulownia tomentosa* Steud (Schneiderova and Smejkal, 2015). It possesses various physiological functions, including anti-bacterial, anti-fungal, and anti-tumor effects (Shaleen and Asmita, 2020; Pereira et al., 2021). In this study, we demonstrated the anticancer effects of paulownin by enhancing NK cell cytotoxicity. The pre-treatment of NK-92 and primary NK cells with paulownin enhanced their cytolytic activity against the K562 human leukemia cell line. We also showed that paulownin significantly increased NK-92 cell cytotoxicity against human lung cancer cell lines (A549 and PC-9) and human colon cancer cell lines (SW480 and HT29). The anticancer efficacy of paulownin was confirmed using a mouse model injected with the B16F10 melanoma cell line, suggesting that paulownin may be a promising candidate for treating various types of cancer. Previous studies have shown that paulownin exerts anticancer efficacy by acting directly on tumor cells. Paulownin showed CC50 values of 70.6 μM and 100 μM against K562 and HeLa cell lines, respectively (Chang et al., 2014; Reis et al., 2022). Here, we demonstrated that paulownin directly increased the cytolytic activity of NK cells at lower doses (5, 10, and 20 μM) against various human cancer cell lines. These enhancements were also observed in paulownin-treated primary NK cells. Furthermore, in the mouse model, treatment with paulownin reduced tumor growth by about 60%. However, when NK cells were removed by anti-asGM, paulownin treatment only decreased tumor growth by about 10%. These results suggest that paulownin suppresses tumor growth, at least in part, by enhancing the cytolytic activity of NK cells.

MAPK and AKT signaling play important roles in NK cell cytotoxicity (Jiang et al., 2000; Ali et al., 2015). Several natural compounds promote the cytolytic activity of NK cells by activating these signaling pathways. For example, resveratrol increased the cytotoxicity of NK cells through AKT and m-TORC2-mediated cMyb upregulation (Lee and Kim, 2020). Metformin promoted the activation of p38 MAPK, which is involved in the secretion of cytolytic granules from NK cells (Xia et al., 2021). 2,3-BTD enhanced the cytolytic activity of human pNK cells and NK-92 cells by increasing JNK and ERK activities (Lai et al., 2012). Here, we investigated the role of AKT and MAPK in paulownin-mediated NK cell cytotoxicity. Our findings showed that paulownin promoted the JNK signaling pathway but not AKT, p38, or ERK signaling. Improvement of NK cell cytotoxicity by paulownin was abolished by the JNK inhibitor. Therefore, although we cannot exclude the involvement of other signaling pathways in the anticancer efficacy of paulownin, the JNK pathway is implicated in paulownin-mediated improvement of NK cell cytolytic activity.

NK cells rapidly release a high concentration of cytolytic granules into the immunological synapse to induce apoptosis of target cells (Alter et al., 2004). CD107a is a functional marker protein of cytotoxic NK cell degranulation, as its surface expression is induced upon degranulation (Aktas et al., 2009). Here, we demonstrated that paulownin promoted the expression of CD107a on NK cells when co-cultured with target cells. Perforin and granzyme B are effector proteins that play crucial roles in NK cell-mediated cytotoxicity (Thiery et al., 2011; Pardo et al., 2002). Interestingly, paulownin significantly increased the expression of perforin while slightly enhancing the expression of granzyme B. Furthermore, JNK inhibitors suppressed the increase in perforin expression caused by paulownin but had no effect on the increase in granzyme B expression. This phenomenon implies that the increased expression of perforin and granzyme B induced by paulownin is caused by different signaling pathways, which warrants further study.

In the present study, we demonstrated the anticancer effect of paulownin through direct activation of NK cell cytotoxicity *in vitro* and *in vivo* for the first time. Our data indicate that paulownin enhances NK cell cytotoxicity by activating the JNK signaling pathway. These findings suggest that paulownin can be a promising candidate for cancer treatment. However, further investigation is necessary to fully elucidate other signaling pathways regulated by paulownin and explore its potential therapeutic applications.

## Data Availability

The original contributions presented in the study are included in the article/Supplementary Material, further inquiries can be directed to the corresponding authors.
