# Supplementary material for: Paulownin elicits anti-tumor effects by enhancing NK cell cytotoxicity through JNK pathway activation
Source: Front Pharmacol. 2024 Sep 4;15:1439079. doi: 10.3389/fphar.2024.1439079 (PMC11408334; doi:10.3389/fphar.2024.1439079)
Supplement: Supplementary file 1 [file Table1.docx]

Supplementary Material

| **Supplementary Table 1. Antibody list** |  |  |
| --- | --- | --- |
| Name | catalog number | company |
| p44/42 MAPK (Erk1/2) (137F5) Rabbit mAb | 4695 | CST |
| Phospho-p44/42 MAPK (Erk1/2) (Thr202/Tyr204) (D13.14.4E) XP® Rabbit mAb | 4370 | CST |
| p38 MAPK (D13E1) XP® Rabbit mAb | 8690 | CST |
| Phospho-p38 MAPK (Thr180/Tyr182) (D3F9) XP® Rabbit mAb | 4511 | CST |
| SAPK/JNK Antibody | 9252 | CST |
| Phospho-SAPK/JNK (Thr183/Tyr185) (81E11) Rabbit mAb | 4668 | CST |
| Akt (pan) (C67E7) Rabbit mAb | 4691 | CST |
| Phospho-Akt (Ser473) (D9E) XP® Rabbit mAb | 4060 | CST |
| β-actin | sc-47778 | Santa Cruz |
| anti-perforin 1 antibody | sc-136994 | Santa Cruz |
| anti- Granzyme B antibody | 4275S | CST |
| APC anti-human CD337 (NKp30) | 325210 | biolegend |
| PE anti-human CD336 (NKp44) | 325108 | biolegend |
| PE anti-human CD335 (NKp46) | 331908 | biolegend |
| PE anti-human CD314 (NKG2D) | 320806 | biolegend |
| BD Pharmingen™ Alexa Fluor 647 mouse anti-human perforin | 563576 | BD |
| BD Pharmingen™ Alexa Fluor® 647 Mouse Anti-Human Granzyme B | 561999 | BD |
| PE anti-human perforin antibody | 353304 | biolegend |
| APC anti-human CD56 | 318310 | biolegend |
| FITC anti-human CD107a | 328606 | biolegend |
| FITC anti-human/mouse Granzyme B recombinant antibody | 372204 | biolegned |
| APC-anti-mouse CD3 antibody | 349201 | BD |
| [BD Pharmingen™ PE Rat Anti-Mouse CD335 (NKp46)](https://www.bdbiosciences.com/ko-kr/products/reagents/flow-cytometry-reagents/research-reagents/single-color-antibodies-ruo/pe-rat-anti-mouse-cd335-nkp46.560757) | 560757 | BD |
