# Supplementary material for: Paulownin elicits anti-tumor effects by enhancing NK cell cytotoxicity through JNK pathway activation
Source: Front Pharmacol. 2024 Sep 4;15:1439079. doi: 10.3389/fphar.2024.1439079 (PMC11408334; doi:10.3389/fphar.2024.1439079)
Supplement: Supplementary file 2 [file DataSheet1.pdf]

Supplementary Figure 1

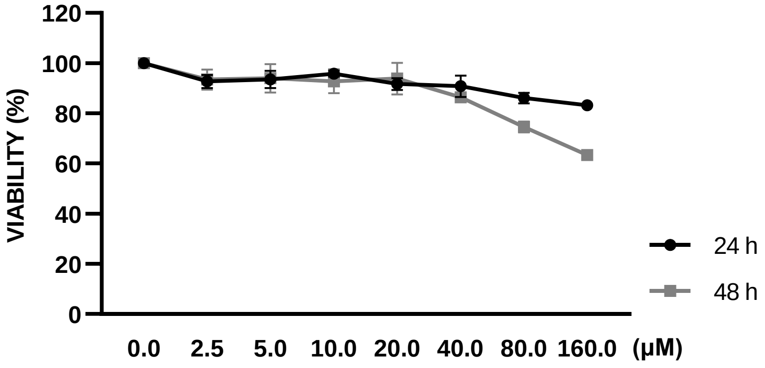

Supplementary Figure 1. The effect of paulownin on viability of NK-92 cells. NK-92 cells were treated with indicated concentrations (0, 5, 10, 20, 40, 80, and 160 μM) of paulwonin for 24 h or 48 h. The viability was measured using the cell counting kit (CCK)-8 assay.

Supplementary Figure 2

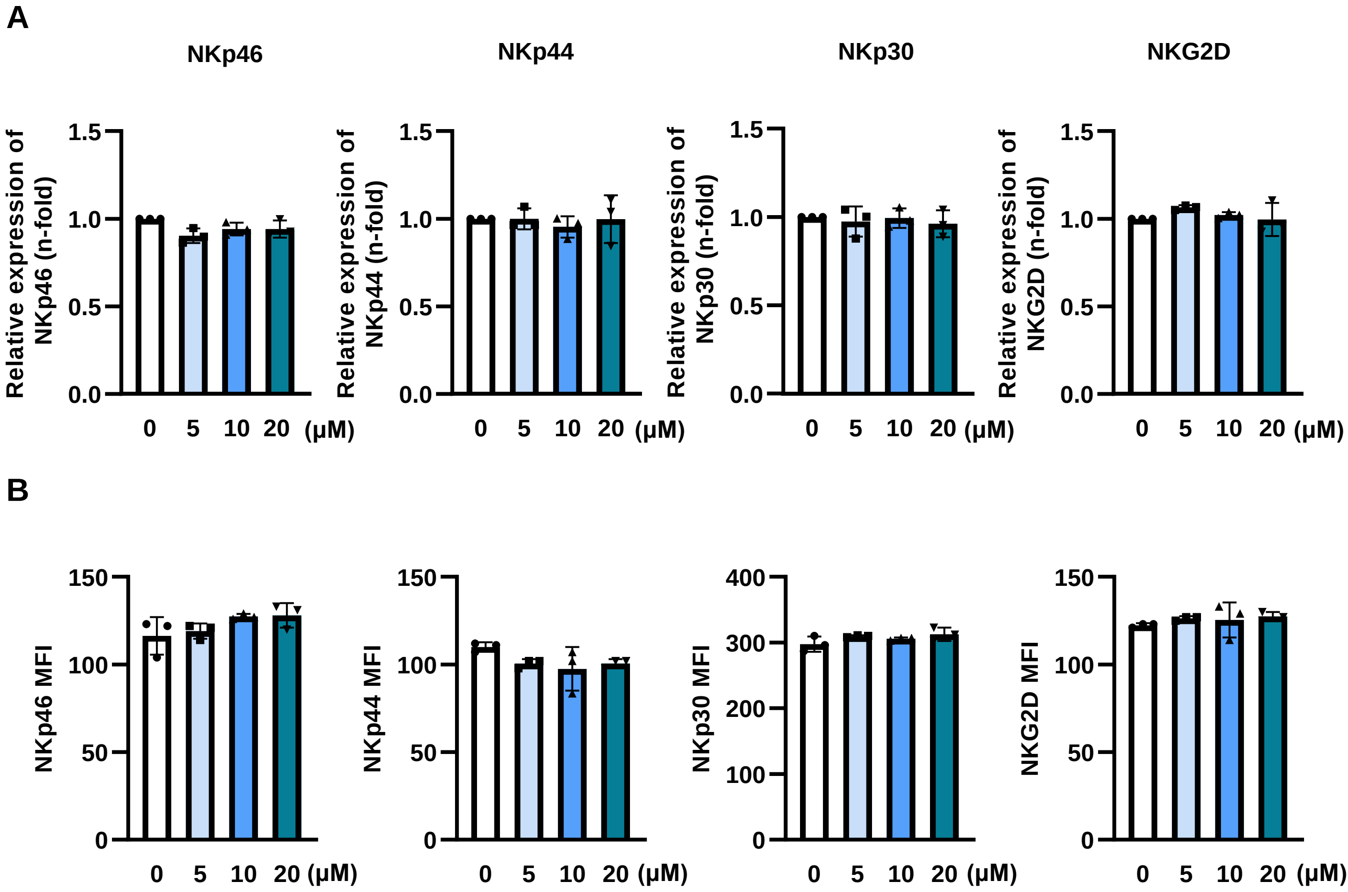

Supplementary Figure 2. Paulownin does not increases the expression of NK activating receptors. NK-92 cells were treated with paulownin (0, 5, 10 and 20 μM). (A) The expression level of mRNA for NKp46, NKp44, NKp30, and NKG2D were detected by qPCR and protein levels were analyzed by flow cytometry. The numbers represent the MFI.

Supplemantary Figure 3

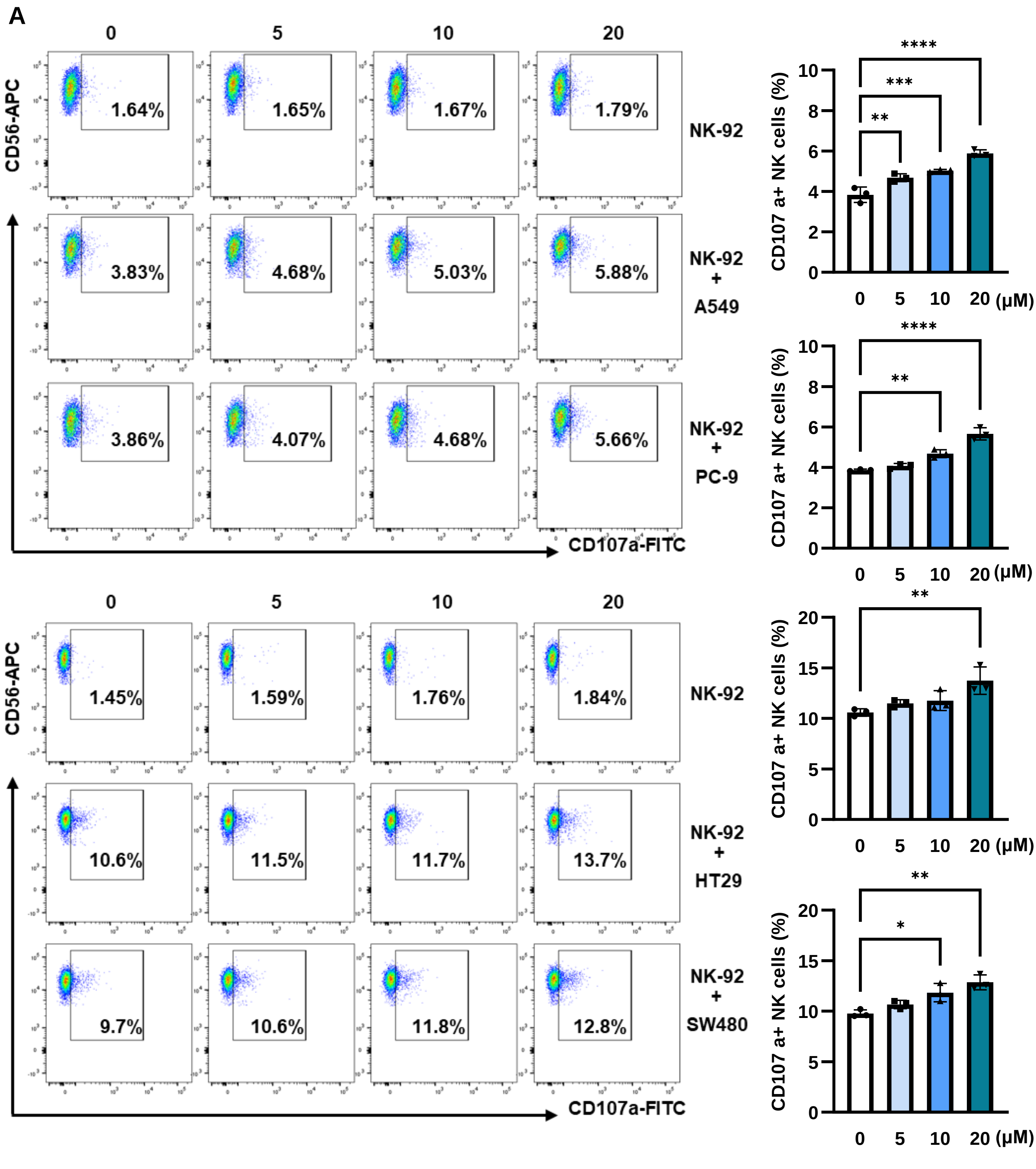

Supplementary Figure 3. Paulownin enhances the cytolytic granules of NK-92 cells against various target cells. (A) Paulwonin pre-treated NK-92 cells were co-incubated with each target cell at a 1:1 ratio for 4h and degranulation of NK-92 cells surface was examined by CD107a expression levels using flow cytometry. Representative dot plots and statistical data show positive frequencies of CD56 + CD107a+ cells. The results represent the mean ± SD of three experiments (*$p < 0.05$, **$p < 0.01$, ***$p < 0.001$, ****$p < 0.0001$ by Student's t-test, n=3).

Supplementary Figure 4

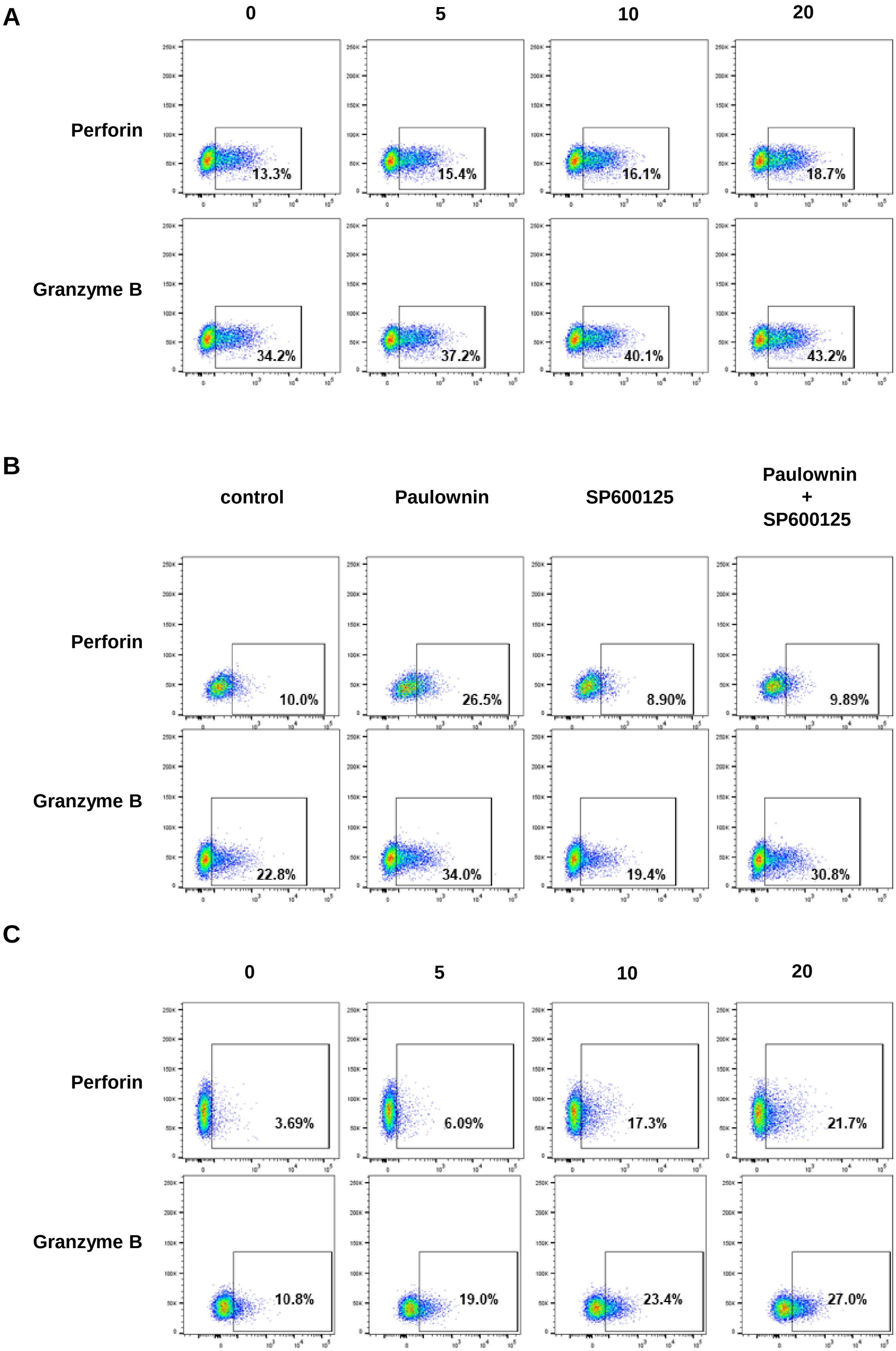

Supplementary Figure 4. Flow cytometry dot plots. Flow cytometry was used to analyze representative dot plots collected from three replicate experiments.

Supplementary Figure 5

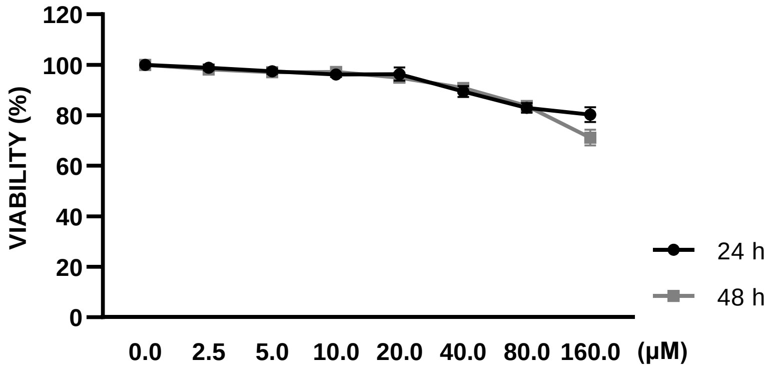

Supplementary Figure 5. The effect of paulownin on viability of primary NK cells. pNK cells were treated with indicated concentrations (0, 5, 10, 20, 40, 80, and 160 μM) of paulwonin for 24 h or 48 h. The viability was measured using the cell counting kit (CCK)-8 assay.
